# Address to the Graduates of Geneva College

**Published:** 1847-04

**Authors:** Charles Alfred Lee

**Affiliations:** Professor of General pathology and Materia Medica in Geneva Medical College, etc., etc.


10. *Address to the Graduates of Geneva Medical College, delivered January 26th*, 1847. By Charles Alfred Lee, A. M., M. D., Professor of General pathology and Materia Medica in Geneva Medical College, etc., etc. Published by request of the Graduates.

This is an able address, and admirably suited to the occasion on which it was delivered. The valedictory address of a professor to his pupils, is almost necessarily monitory in its character. It is like the parting words of a parent to his son, pointing to his moral responsibilities and the great ethical rules by which he should be governed, and hence we rarely look for anything original, argumentative or ingenious; but it is something to express ordinary truths in language befitting the occasion, and calculated to win the attention, engage the affections, and persuade the judgment of the hearers. In these respects Dr. Lee has certainly been successful. We have room for only one or two extracts, which we select, not for any novelty in either the subjects or the sentiments, but for their importance an truthfulness.—*Ibid.*

"For some time after commencing your professional life, you will probably have some leisure on your hands, which you can turn to profitable account by devoting it to study. Be not discouraged at the want of speedy success; your merits will eventually be known, and you will be rewarded accordingly. Justice will sooner or later be done you, and if you aim at eminence, and your efforts are well directed, you will attain it. Aim first at the establishment of character and reputation, with the full assurance that all desirable consequences will follow in their train. Turn not aside into any of the devious, albeit fashionable, paths of quackery so rife at the present day, by whatever specious name they may be known; sacrifice not your prospects and your good name by becoming the adherents of any partial and exclusive systems of medicine, for you may rest assured that they will all speedily disappear 'like the baseless fabric of a vision.' Belittle not your honorable title of PHYSICIAN by prefixing to it any distinctive or diminutive epithet, be it Thomsonian, Homœopathic, or Hydropathic; for why should you do this? Is not he who stands upon the broad platform of catholic medicine more likely to be better armed for attacking disease, than he who occupies some insignificant redoubt, or petty loop-hole? Is there any want of freedom of opinion in our profession? Is not every one at liberty to construct his own articles of faith, drawing from every system whatever portion of truth it may contain, and shape his practice accordingly? Medicine is not, as many seem to suppose, a system of rules and doctrines handed down from teacher to pupil, admitting no change, a set of formulæ which you are bound to sustain, and from which it were heresy to swerve; but it is a progressive and constantly improving science, and every true and sincere votary of it will employ *all* the remedies, means, and resources within his reach, which accident or science has discovered, and observation and experiment verified. Away, then, with your partial systems which inevitably and professedly limit these means, and virtually nullify these resources. There is, indeed, gentlemen, a sad relaxation of principle at the present day, even in some who are regarded as among the most distinguished members of the profession, as manifested by their patronage and recommendation of patented and secret remedies; a course of conduct which is obviously incompatible with every sentiment of moral duty, and every principle of sound medical ethics. To keep from the world any discovery calculated to benefit mankind, as connected with the preservation of human health, or its restoration when lost, is such a derilection of duty, as to have met with the reprobation of the wise and good in every age of the world; and when this is done, as it generally is, for the sake of pecuniary emolument, the mind instinctively revolts at it, as an exhibition of selfishness and insensibility to human suffering disgraceful to our natures, and derogatory to the character of those who belong to a profession, whose foundation is philanthropy, and whose crowning glory is benevolence. Give not, then, the slightest encouragement to remedies of this description, or their inventors; frown indignantly upon all attempts to render our glorious art a mercenary trade; disgrace not your *Alma Mater* and your own reputation, by countenancing, in the slighest degree, any unworthy proceedings of this kind, for, by so doing, you will justly forfeit all title to respect, and take rank with the Brandreths and Moffats of the day. The time is not distant when such a deep stain of disgrace must inevitably attach to patentees and proprietors of secret remedies in our profession, that neither the waters of Lethe will be able to obliterate nor the exhibition of 'Letheon' to bury in oblivion.

"Should any of you, then, hereafter discover a remedy calculated to benefit the world, publish it upon the house-top—imitate the goodness of Providence, and make it free as the air we breathe; for the consciousness of having done a good deed for humanity, the gratitude of an intelligent community, and the praises of a liberal profession, shall prove a most satisfactory reward.

"There is one duty which you owe to yourselves, to the sick who may be entrusted to your charge, and to society, by whose favor and confidence you are to be sustained, and this is—to shun the use of intoxicating drinks. I urge this upon you, Graduates of Geneva College, by every consideration of duty, of honor, of interest, and of philanthropy; I charge you as you value reputation, usefulness, success, and an approving conscience, ever adhere to the strictest rules of temperance. You owe this to those who have sustained you thus far, and furnished you with the means of obtaining a medical education; you owe it to your teachers who have labored to instruct you in the various branches of your art, and who feel an anxious desire for your prosperity; you owe it to the beloved Alma Mater, who sends you forth with pride this day, to carry health, and virtue, and gladness to those who come within your influence; you owe it to the profession whose bright escutcheon must never be soiled by your example; you owe it to society in whose ranks you are now to be enrolled, we trust among its most valued and respected members; you owe it to your hopes of usefulness here, and of happiness hereafter; you owe it to a reformed and enlightened public opinion; and, lastly, you owe it to your God. As you go forth, then, upon the serious errand of your lives—an errand requiring the keen eye, the cool head, the steady hand, the sound judgment—take this, my solemn and affectionate warning, along with you; carry it into the social circle and the recesses of private life; take it into the hospitable mansions of the rich and the lowly dwellings of the poor; remember it in the hour of temptation and trial; heed it when the syren voice of pleasure beckons you along her flowery paths; so shall your lives flow equitably along; your cup of happiness be filled; your days crowned with usefulness, and your names with honor."

